# Supplementary material for: Disruption of TFIIH activities generates a stress gene expression response and reveals possible new targets against cancer
Source: Open Biol. 2020 Jun 17;10(6):200050. doi: 10.1098/rsob.200050 (PMC7333893; doi:10.1098/rsob.200050)
Supplement: supplementary material- Figures [file rsob200050supp1.docx]

**ELECTRONIC SUPPLEMENTARY MATERIAL**

**1. Molecular dynamics methods**

*II.1.1. Model preparation*

The cryo-electron microscopy (cryo-EM) structure of *Homo sapiens* TFIIH (PDB ID: 6NMI^1^) was retrieved from the Protein Data Bank (PDB). Not solved side-chains were completed with WHAT-IF server^2^. The missing loops were modelled using MODELLER v9.17^3^. For covalent molecular docking and molecular dynamics (MD) simulations, only the XPB-p52-p8 submodule of the TFIIH complex was used. Figure S3*a* depicts a tridimensional structure of the submodule coloring the XPB component by its four domains: N-terminal domain (NTD), DNA recognition domain (DRD) and helicase domains (HD1 and HD2). The highly flexible loop of XPB that interacts with MAT1 (aa 200 to 265) was omitted during the MD to avoid artifacts during the simulations.

*II.1.2. Covalent molecular docking*

*Ligand preparation*. A preoptimized 3D conformer of TPL was retrieved from the PubChem database^4^. Based on previous TPL reactive sites reported by He *et al.*^5^, a total of five TPL-Cys complexes were constructed by covalently linking the sulfur of the cysteine with each of the following carbons: C4, C7, C9, C11 and C12 (see Supplementary Fig S3a). The five TPL-Cys complexes were optimized using the MMFF94 force field (mmff94) implemented in the *obminimize* module of the OpenBabel toolbox^6^. A further geometry optimization of the complexes were carried out with the B3LYP functional as implemented in Gaussian 09 package.^7,8^ Finally, Gasteiger-Marsili atomic partial charges and AutoDock atom types were assigned with the MGLTools v1.5.6 package^9^ for the PDBQT generation.

*Protein preparation.* Polar hydrogens, Gasteiger-Marsili atomic partial charges and AutoDock atom types were computed for the XPB-p52-p8 submodule employing the MGLTools v1.5.6 package.^9^

*Protein-Ligand preparation.* Each TPL-Cys complex was oriented relative to the position of Cys342 residue located in the XPB subunit of the submodule and assigned as a flexible residue of the protein.

*Molecular docking*. The oriented TPL-Cys complexes were docked into XPB using the covalent docking protocol of AutoDock v4.2 software.^10^ First, covalent docking of the TPL was performed in Cys352 taking into account the flexibility of residues located in the first (TBS1: W493, Q497, Q638, Q683, Y685) and second (TBS2: K346, N366, Q373, E442, Q638, R642) possible TPL binding sites (TBSs, Supplementary Fig S3a). For each complex, a total of 50 runs were performed with a maximum number of 25,000,000 energy evaluations and 27,000 generations using the Lamarckian genetic algorithm as a search method. The most representative conformations of the binding site residues were clustered and employed for a second covalent docking using the same search parameters but giving flexibility only to TPL-Cys complexes. The latter was carried out to define the docking score of each of the resulting XPB-TPL complexes from considering different orientations of the residues in the TBSs (Supplementary Table S5).

*XPB-TPL selection*. The covalent docking results suggest that the formation of the Cys-TPL complex is less likely to occur at the C9 position of TPL due to the high score values recorded for this reactive site in both TBSs. For TBS1, we observed that TPL has a high probability of forming a stable complex by reacting with Cys342 at positions C7, C11 and C12. On the other hand, in the TBS2 site TPL has the probability of forming the covalent bond with any of the five positions. A study conducted by He *et al.*^5^ demonstrated, using different XPB variants and TPL analogues, that the C12 site of TPL (TPL_C12_) is the one that forms the covalent bond with Cys342. Our covalent molecular docking results, along with this experimental data, suggest that the TPL forms a covalent bond with Cys342 through its C12 reactive site and has two possible binding sites at the HD1-HD2 interface of XPB (Supplementary Fig S3b). We extracted the best ranked binding modes of TPL_C12_-Cys for the construction of the protein-ligand complexes with the XPB-p52-p8 submodule.

*II.1.3. Molecular dynamics simulations*

The apo and the two TPL-bound structures of XPB-p52-p8 submodule were evaluated through 100 ns of atomistic MD simulations using the AMBER99SB-ILDN force field^11^ implemented in the GROMACS 5.1.4 package^12^. Topology and TPL_C12_-Cys atom types were parametrized in the amber framework with the ACPYPE interface^13^ and included in the GROMACS parameter files to be recognized as an amino acid. The three models were solvated using a TIP3P water cube and neutralized with a NaCl solution 0.15 M. The systems were submitted to a mild minimization using the *steepest descent* and *conjugate gradient* algorithms. Following the minimizations, each system was heated to 300 K and equilibrated for 1,000 ps in a canonical ensemble (NVT) using the Nosé-Hoover thermostat^14^. The systems were further equilibrated for 1,000 ps at 1 bar in a isothermal-isobaric ensemble (NPT) with the Parrinello-Rhaman barostat^15^. Finally, each system was submitted to 100 ns non-restrained NPT production runs with a 0.002 ps timestep and recorded every 5 ps. The neighbor list was updated every 20 steps and non-polar hydrogen bonds were constrained using the LINCS algorithm^16^. The Lennard-Jones potential was set using a shift function between 1.0 and 1.2 nm, and electrostatic interactions were calculated within a cutoff radius of 1.2 nm. The long ranges electrostatic interactions were calculated with the particle mesh Ewald (PME) method using a Fourier grid spacing of 1.2 nm.^17^

From each MD simulation, we calculated the backbone root-mean-square deviations (RMSD, Supplementary Fig S3c) and the number of contacts formed between the HD1 and HD2 domains of XPB (Num of Contacts*,* Supplementary Fig S3d) using the GROMACS built-in tools. A RMSD-based clustering analysis showed that TPL, initially placed in the TBS1 site, was shifted to TBS2 during the simulation (Supplementary Fig S3e). The latter suggests that there is a greater probability that TPL is oriented towards TBS2 than to TBS1. Finally, we calculated the occupied fraction of HD1 and HD2 residues located within 3.5 Å of TPL and between the each other (Supplementary Fig 3f and S3g). The 3D figures and plots were generated with PyMOL v0.9^18^ and Gnuplot 5.0^19^, respectively.

**References**

1. Greber, B. J., Toso, D. B., Fang, J. & Nogales, E. The complete structure of the human TFIIH core complex. *Elife* **8**, 1–29 (2019).

2. Vriend, G. WHAT IF: a molecular modeling and drug design program. *J. Mol. Graph. Model.* **8**, 52–56 (1990).

3. Webb, B. & Sali, A. Comparative protein structure modeling using MODELLER. *Curr. Protoc. Bioinforma.* **47**, 5.6.1-5.6.32 (2014).

4. National Center for Biotechnology Information. PubChem Compound Database. (2017).

5. He, Q. L. *et al.* Covalent modification of a cysteine residue in the XPB subunit of the general transcription factor TFIIH through single epoxide cleavage of the transcription inhibitor triptolide. *Angew. Chemie - Int. Ed.* (2015). doi:10.1002/anie.201408817

6. O’Boyle, N. M. *et al.* Open Babel: An Open chemical toolbox. *J. Cheminform.* **3**, 1–14 (2011).

7. Frisch, M. J. . *et al.* Gaussian 09, Revision E.01. *Gaussian, Inc., Wallingford CT* (2009).

8. Zhu, S., Yan, L., Ji, X. & Lu, W. Conformational diversity of anthracycline anticancer antibiotics: A density functional theory calculation. *J. Mol. Struct. THEOCHEM* **951**, 60–68 (2010).

9. Morris, G. M. *et al.* AutoDock4 and AutoDockTools4: Automated docking with Selective Receptor Flexibility. *J. Comput. Chem.* **30**, 2785–2791 (2009).

10. Bianco, G., Forli, S., Goodsell, D. S. & Olson, A. J. Covalent docking using autodock: Two-point attractor and flexible side chain methods. *Protein Sci.* **25**, 295–301 (2016).

11. Lindorff-Larsen, K. *et al.* Improved side-chain torsion potentials for the Amber ff99SB protein force field. *Proteins Struct. Funct. Bioinforma.* **78**, 1950–1958 (2010).

12. Abraham, M. J. *et al.* GROMACS: High performance molecular simulations through multi-level parallelism from laptops to supercomputers. *SoftwareX* **1**–**2**, 19–25 (2015).

13. Sousa da Silva, A. W. & Vranken, W. F. ACPYPE - AnteChamber PYthon Parser interfacE. *BMC Res. Notes* **5**, 367 (2012).

14. Braga, C. & Travis, K. P. A configurational temperature Nosé-Hoover thermostat. *J. Chem. Phys.* **123**, 0–15 (2005).

15. Parrinello, M. & Rahman, a. Strain fluctuations and elastic constants. *J. Chem. Phys.* **76**, 2662 (1982).

16. Hess, B., Bekker, H., Berendsen, H. J. C. & Fraaije, J. G. E. M. LINCS: A linear constraint solver for molecular simulations. *J. Comput. Chem.* **18**, 1463–1472 (1997).

17. Darden, T., York, D. & Pedersen, L. Particle mesh Ewald: An N⋅log(N) method for Ewald sums in large systems. *J. Chem. Phys.* **98**, 10089 (1993).

18. DeLano, W. L. The PyMOL Molecular Graphics System, DeLano Scientific LLC,Palo Alto, CA. http://www.pymol.org (2007).

19. Williams, T. & Kelley, C. Gnuplot: an interactive plotting program. (2016).

**2. Supplementary Figures**

**
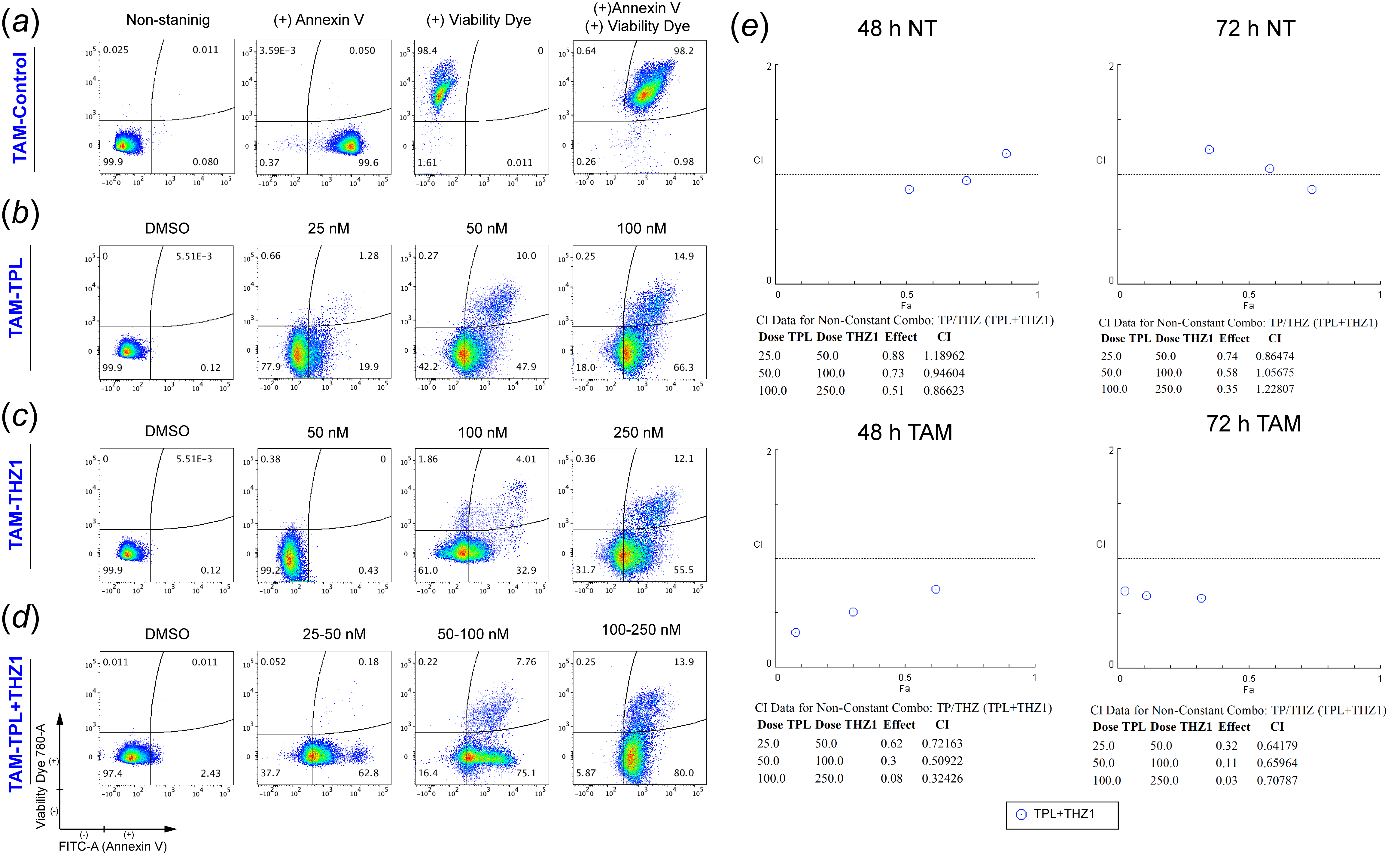
**

**Figure S1.** Triptolide (TPL), THZ1, and TPL/THZ1 combinatory, treatments induce apoptosis in MCF10A-ErSrc cells. Apoptosis was determined by Annexin-FITC (x-axis) and cell viability by Viability Dye 780 (y-axis). (*a*) TAM cells used as a control to define the quadrants. (*b*) TAM cells treated with TPL for 72 h at 25, 50 and 100 nM. (*c*) TAM cells treated with THZ1 for 72 h at 50, 100 and 250 nM. (*d*) TPL/THZ1 combinatory treatment, 25/50, 50/100 and 100/250 nM of each substance. Plots showed as a representative example from three biological replicates. (*e*) Drug combination index (CI) with respect to the fraction affected (Fa) for the viability effect of TPL+THZ1 calculated with Chou-Talalay equation using CompuSyn software.

**
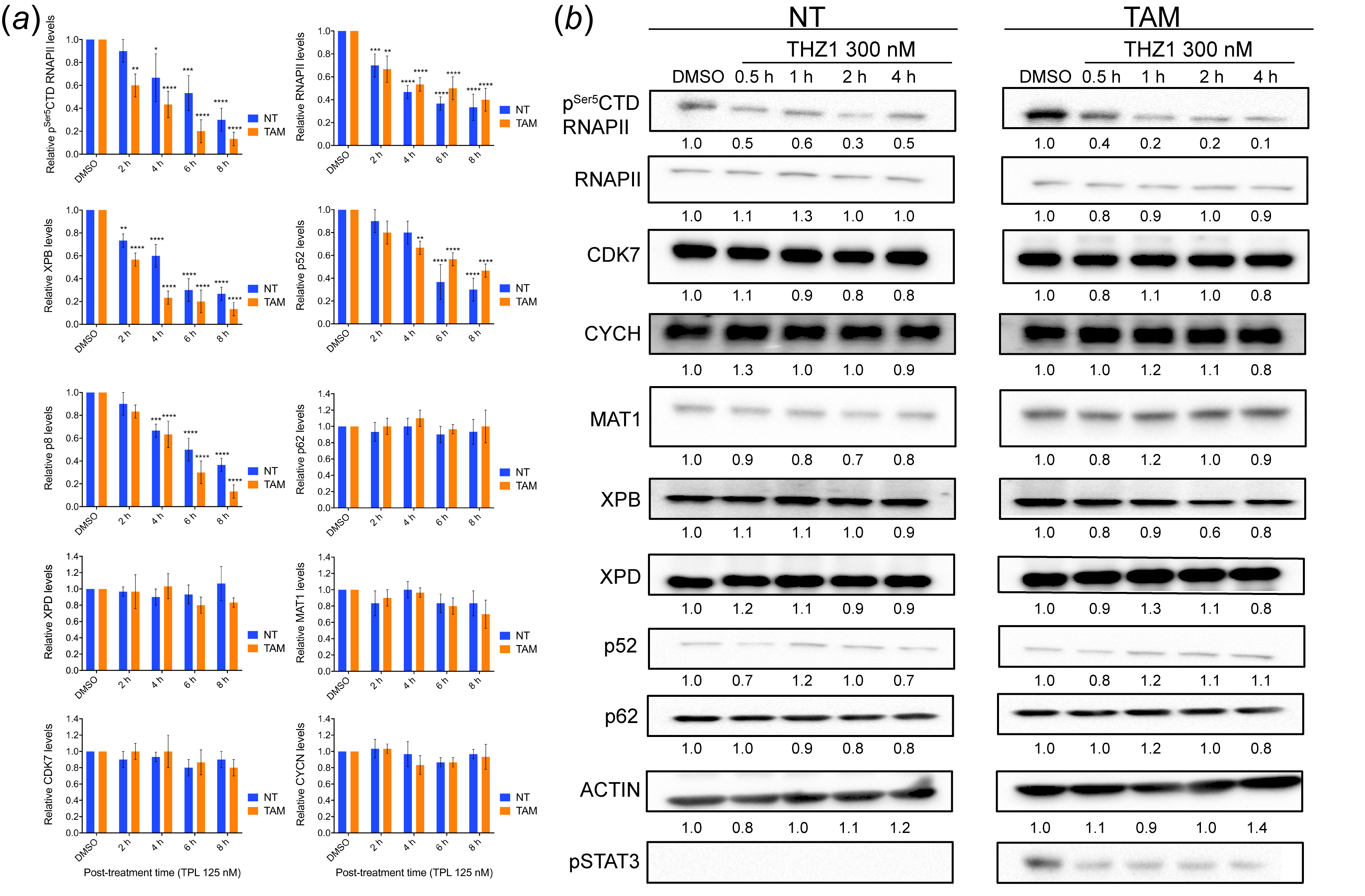
**

**Figure S2.** Effect of TPL and THZ1 on TFIIH. (*a*) Densitometric analysis of RNAPII, p^Ser5^CDT RNAPII, and some TFIIH subunits (XPB, p52, p8, p62, XPB, MAT1, CDK7, and CYCH) normalized against tubulin or actin protein levels.  The graphs show mean values ± SD (Standard Deviation) of three biological replicates. Significant differences were analyzed by Two-way ANOVA with corrections for multiple comparisons, always comparing with the DMSO column. Statistical significance is indicative **P<0.01, ***P<0.001 or ****P<0.0001. (*b*) Tamoxifen-treated (TAM) and Non-Treated (NT) cells incubated with THZ1 (300 nM) at 0.5, 1, 2 and 4 h in comparison with the control with DMSO for 4 h. The levels of RNAPII, p^Ser5^CDT RNAPII, and some TFIIH subunits (CDK7, CYCH, MAT1, XPB, XPD, p52, and p62) were evaluated. The p-STAT3 is used as control of transformation in the line. Densitometric analyses were performed using actin as loading control; the relative quantification is indicated under each blot and the data showed as a representative example from three biological replicates.

**

**

**Figure S3.** XPB-p52-p8 submodule of TFIIH and TPL interactions. (*a*) Depiction of the XPB-p52-p8 submodule tridimensional structure, the XPB component is colored by its domains: NTD (blue), DRD (green), HD1 (yellow) and HD2 (red). TPL chemical structure at the bottom shows the carbon numbering of the five potential attack sites by C342. Panel on the right shows the amino acid residues that constitute the TBS1 (upper) and TBS2 (bottom) at the HD1-HD2 interface of XPB. (*b*) Best-ranked binding poses of TPL covalently bound to C342 residue at the two possible binding sites: TBS1 (uppper) and TBS2 (bottom). (*c*) The backbone root-mean-square deviations (RMSD) of apo (black) and the TBS1 (light purple) and TBS2 (dark purple) TPL-bound structures of XPB subunit. (*d*) Number of contacts formed between the HD1 and HD2 domains of XPB in the apo and the two bound states during the simulation. (*e*) Depiction of TPL shift from TBS1 to TBS2 through 100 ns MD simulation. (*f*) Occupied fraction of HD1 and HD2 residues located within 3.5 Å of TPL during the last 80 ns of the simulated time. (*g*) Occupied fraction of HD1 residues located within 3.5 Å of HD2 and HD2 residues located within 3.5 Å of HD1.

| **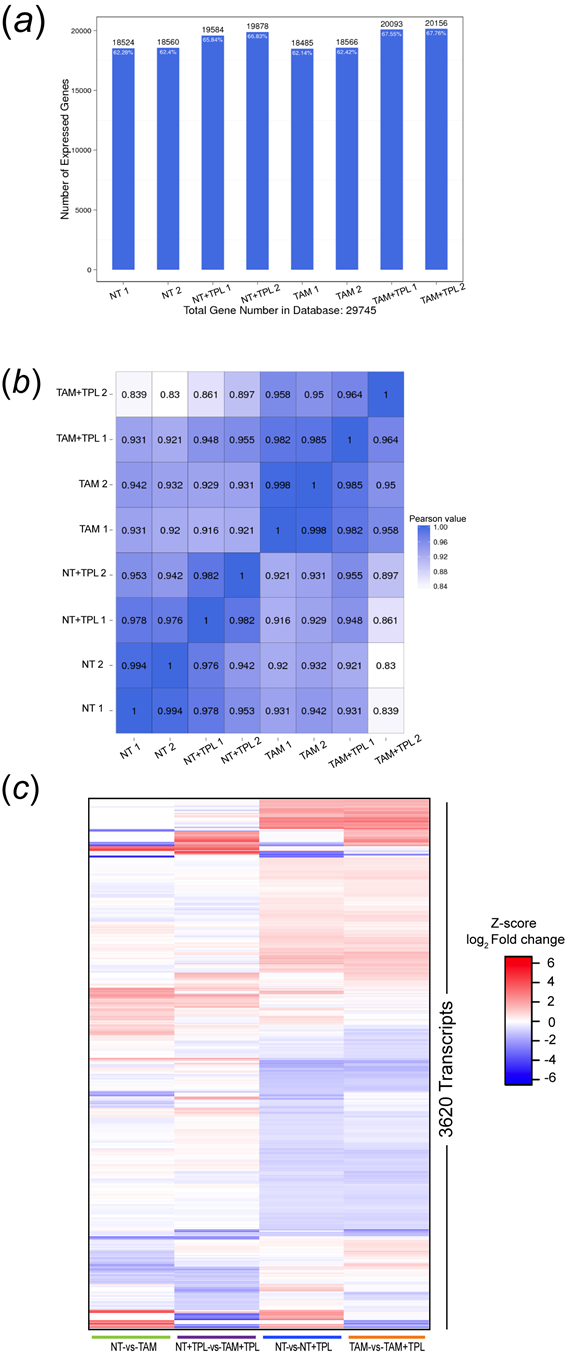** | **Figure S4.** Global correlation between the expressed genes obtained in the transcriptional analysis. (*a*) Number of genes identified in the transcriptional analysis, in the x-axis is the sample name and in the y-axis are the identified expressed genes. The proportion at the top of each bar represents the number of expressed genes number divided by the total gene number reported in the database. (*b*) Pearson′s correlation coefficient values across each sample; the barcode colour gradient indicates the minimum value as white and the maximum as blue. If one sample is highly similar to another one, the correlation value between them is very close to 1. (*c*) Genes that were differentially expressed (3620 transcripts) in all pairwise of cluster plans. |
| --- | --- |

**
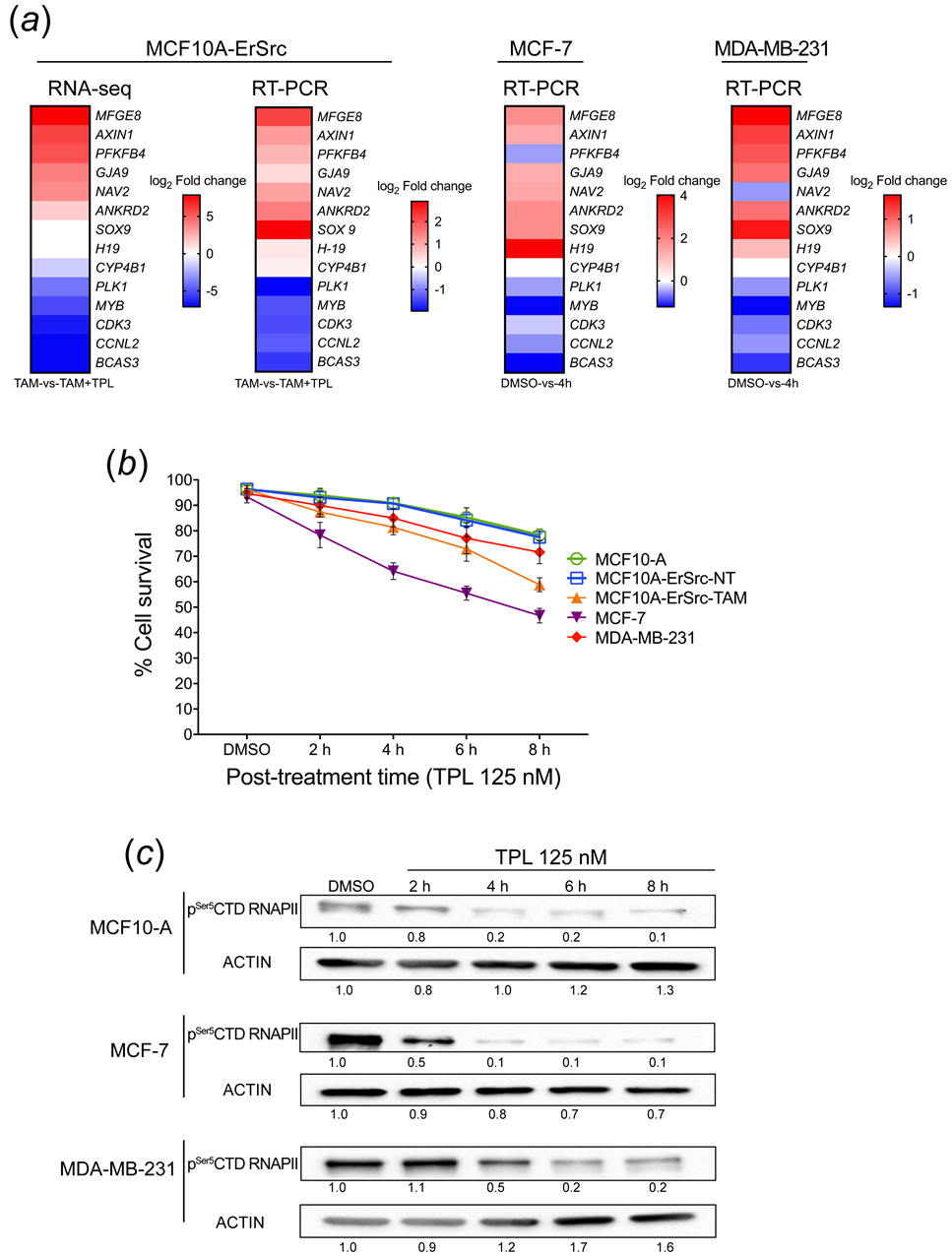
**

**Figure S5.** Transcriptional data of selected genes to corroborate the TPL effect on transformed cells. (*a*) The left panel shows the expression levels from data obtained by the RNA-Seq. The central panel shows the expression levels of the same genes verified by RT-PCR and right panel shows the expression levels of the same genes analysed in other cell lines (*b*) Cell viability of MCF10-A (green), MCF10A-ERSrc-NT (blue), MCF10A-ERSrc-TAM (orange), MCF-7 (Purple) and MDA-MB-231 (red) cell lines are treated with TPL at 125 nM for 2, 4, 6 and 8 h. The control cells were treated with growth medium or DMSO for 8 h. (*c*) p^Ser5^CTD RNAPII levels in the MCF10-A, MCF-7, and MDA-MD-231 cell lines, after being incubated with TPL. Control cells were incubated with DMSO for 8 h. Densitometric analyses were performed using actin as loading control; the relative quantification is indicated under each blot and the data showed as a representative example from three biological replicates.


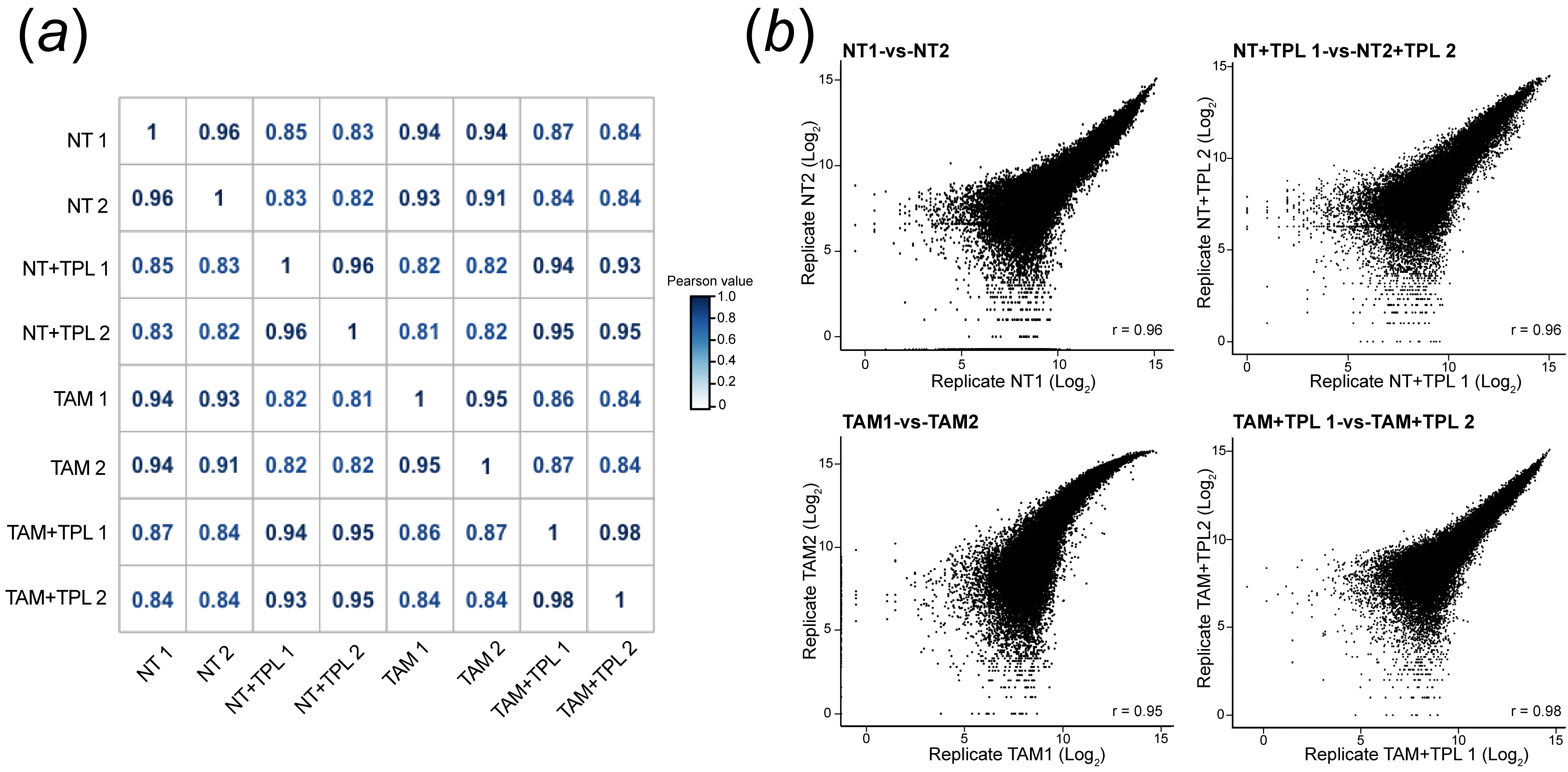


**Figure S6**. Pearson´s correlation between ChIP-Seq replicas. (*a*) Pearson´s correlation coefficient values across each sample. Tamoxifen-treated (TAM) and Non-Treated (NT) cells were incubated with TPL 125 nM for 4 h; the correlation value between them is close to 1. (*b*) Correlation graphs between the ChIP-Seq samples of two biological replicates were compared by calculating signal at all the promoters and the bodies of the genes.

| 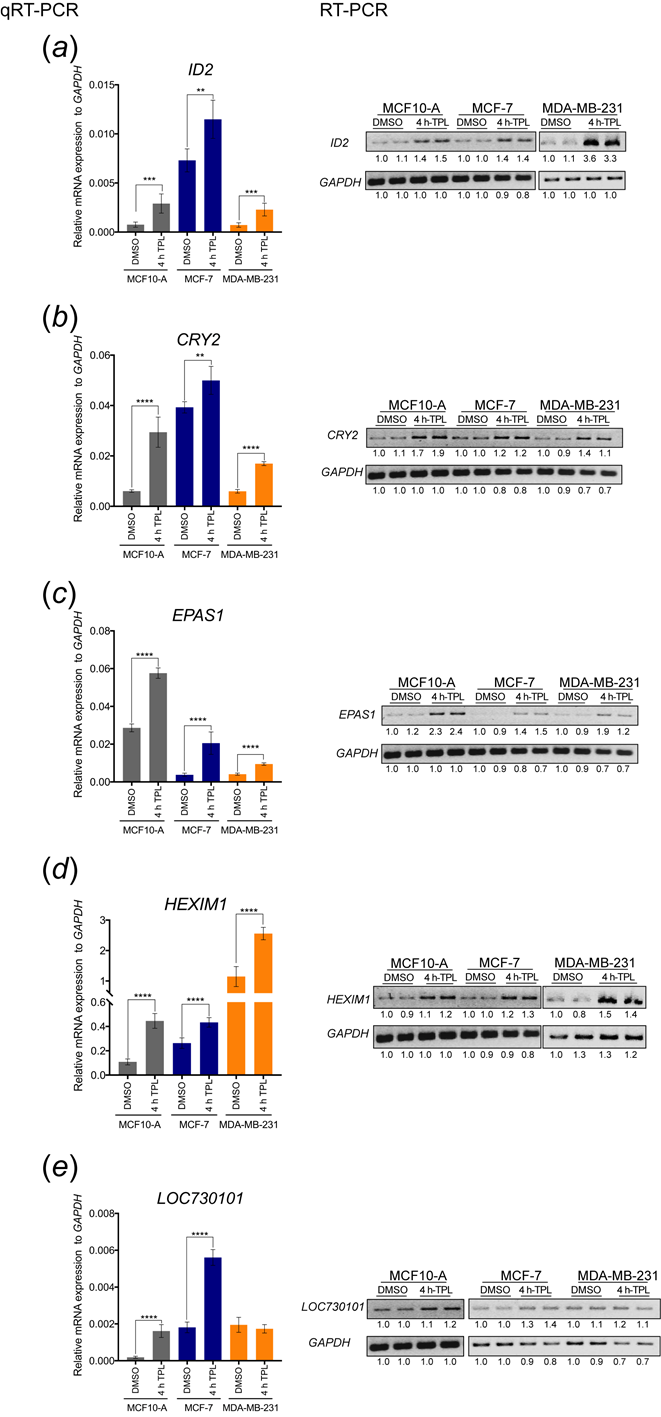 | **Figure S7.** Analysis of genes overexpressed in response to TPL in other breast cancer cell lines. Quantitative RT-PCR and RT-PCR (left and right respectively) analysis of the (*a*) *ID2*, (*b*) *CRY2*, (*c*) *EPAS1*, (*d*) *HEXIM1* and (*e*) *LOC730101* genes in the MCF10A, MCF7 and MDA-MB-231 cell lines. Densitometric analyses were performed using *GAPDH* as a loading control and the relative quantification is indicated under each blot. Graphs show mean values ± SD (Standard Deviation). Significant differences were analyzed by *t*-test. Statistical significance is indicated (*****P*<0.0001 or ****P*<0.001). Right and left panel represent at less two and three independent biological samples, respectively. |
| --- | --- |
